# Design and Evaluation of a Lactate Microbiosensor: Toward Multianalyte Monitoring of Neurometabolic Markers In Vivo in the Brain

**DOI:** 10.3390/molecules27020514

**Published:** 2022-01-14

**Authors:** Eliana Fernandes, Ana Ledo, Rui M. Barbosa

**Affiliations:** 1 Faculty of Pharmacy, University of Coimbra, 3000-548 Coimbra, Portugal; eliana.fernandes2604@ff.uc.pt (E.F.); analedo@ff.uc.pt (A.L.); 2 Center for Neuroscience and Cell Biology, University of Coimbra, 3004-504 Coimbra, Portugal

**Keywords:** electrochemical biosensors, carbon fiber microelectrodes, lactate and glucose, insulin, in vivo brain monitoring

## Abstract

Direct in vivo measurements of neurometabolic markers in the brain with high spatio-temporal resolution, sensitivity, and selectivity is highly important to understand neurometabolism. Electrochemical biosensors based on microelectrodes are very attractive analytical tools for continuous monitoring of neurometabolic markers, such as lactate and glucose in the brain extracellular space at resting and following neuronal activation. Here, we assess the merits of a platinized carbon fiber microelectrode (CFM/Pt) as a sensing platform for developing enzyme oxidase-based microbiosensors to measure extracellular lactate in the brain. Lactate oxidase was immobilized on the CFM/Pt surface by crosslinking with glutaraldehyde. The CFM/Pt-based lactate microbiosensor exhibited high sensitivity and selectivity, good operational stability, and low dependence on oxygen, temperature, and pH. An array consisting of a glucose and lactate microbiosensors, including a null sensor, was used for concurrent measurement of both neurometabolic substrates in vivo in the anesthetized rat brain. Rapid changes of lactate and glucose were observed in the cortex and hippocampus in response to local glucose and lactate application and upon insulin-induced fluctuations of systemic glucose. Overall, these results indicate that microbiosensors are a valuable tool to investigate neurometabolism and to better understand the role of major neurometabolic markers, such as lactate and glucose.

## 1. Introduction

Neuronal activity linked to chemical neurotransmission is a highly energy demanding process and depends on continuous glucose–oxygen supply from blood circulation [1,2,3]. Traditionally viewed as a metabolic byproduct of glucose metabolism, a number of studies have demonstrated that neurons can utilize lactate as a metabolic substrate under fully aerobic conditions, which is accompanied by an increase in extracellular lactate following sustained neuronal activity [4,5].

The preferential utilization of glucose or lactate under resting conditions and neuronal activation has been a matter of debate due to inconsistent results obtained in different experimental models and conditions [6]. Difficulties can, in part, be attributed to the heterogeneous nature of the cellular organization of the brain and the limited spatial and temporal resolution of the analytical techniques that have been more frequently used to study neurometabolism. Therefore, there is a need for developing reliable and robust biosensors tools for monitoring rapid and transient neurochemical changes, including the trafficking of lactate and glucose in the brain extracellular fluid at resting and following neuronal activation. When compared to current standard techniques, such as noninvasive PET or invasive brain microdialysis, the use of biosensors based on microelectrodes coupled to high speed electrochemical techniques offers important advantages regarding spatial, temporal, and chemical resolutions [7,8,9,10,11].

In recent decades, carbon fiber microelectrodes (CFM) associated with fast electrochemical techniques have been extensively used for the real time detection of neurochemicals, mostly due to their unique electrochemical properties, biocompatibility, and minimal tissue damage [12,13,14,15,16].

The non-electroactive nature of glucose and lactate prevents the use of bare CFM to directly probe such metabolic substrates in the brain tissue [15]. Usually, the rational design of microbiosensors is accomplished by immobilizing oxidase enzymes in a polymeric film on a suitable electrode surface, such as Pt, combined with the amperometric detection of hydrogen peroxide (H_2_O_2_). Therefore, numerous studies have been carried out based on Pt:Ir (90:10) microelectrodes of cylindrical or disk geometry [17]. Alternatively, platinized carbon fiber electrodes have also been used to develop microbiosensors for in vivo monitoring [18,19].

A major disadvantage of using noble metals, such as Pt, is related with the relatively high overvoltage, ca. + 0.7 V vs. Ag/AgCl, required to oxidize H_2_O_2_ on the metalized CFM surface. Ascorbate, which is a prominent electroactive molecule in the brain extracellular fluid (ECF), can act as an interferent at the working potential used for hydrogen peroxide detection. This drawback has been circumvented by coating the electrode surface with permselective membranes of Nafion^®^ [20] and polypyrrole [21]. In particular, the electropolymerization of phenylenediamines (PD) monomers, such as *o*-PD or *m*-PD has been wildly used to minimize interference from large molecules, such as ascorbate [22,23,24,25].

We have previously designed and characterized a microbiosensor based on platinized CFM modified with glucose oxidase for high spatio-temporal glucose measurements in brain slices [26]. In the present work, we aim to further extend our previous work by design and evaluation of the enzyme kinetics and analytical performance of a lactate microbiosensor based on Pt modified CFM with lactate oxidase for in vivo monitoring. The novel lactate microbiosensor was combined with a glucose microbiosensor to perform concurrent monitoring of both neurometabolic substrates in vivo in the rat brain. The concurrent real-time monitoring of these neurometabolic markers is a valuable tool to investigate the neurometabolism and to better understand the played role of major neurometabolic markers in vivo. The feasibility of this approach was demonstrated by successfully monitoring locally applied glucose and lactate into the brain, and by monitoring lactate concurrently with glucose in anesthetized rats following systemic insulin administration.

## 2. Results and Discussion

### 2.1. Platinization of Carbon Fiber Microelectrodes

The platinum CFM electrodeposition was performed by potentiostatic electroreduction of PtCl_6_^2^^−^ on the exposed carbon surface tip, as we previously described [26]. Based on H_2_O_2_ sensitivity, the potentiostatic method demonstrated high efficiency in loading Pt onto the electrode surface tip. The size and density of the Pt particles on the CFM active tip surface resulted in an electrochemical behavior similar to bulk Pt or Pt-Ir (90:10) wires commonly used in the brain tissue for measuring neurochemicals [26,27,28].

### 2.2. Electrochemical Behavior of CFM/Pt Microelectrodes

#### 2.2.1. Acidic Electrolyte

**Figure 1:**
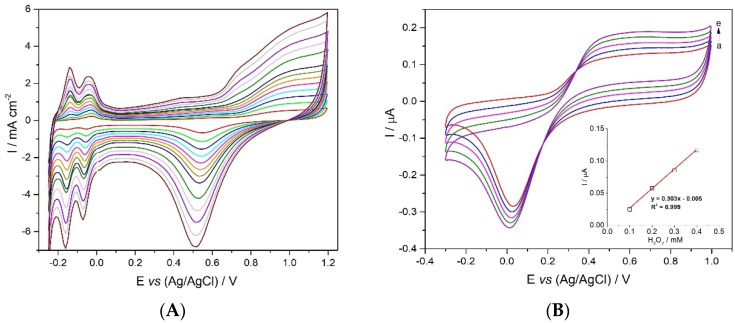
Electrochemical behavior of the CMF/Pt in acidic and neutral electrolyte media. (**A**) Successive cyclic voltammograms (25th scan) at increasing scan rates (50–1000 mV s^−1^) obtained in deoxygenated H_2_SO_4_ (0.5 M); (**B**) Cyclic voltammograms between −0.3 and + 1.0 V vs. Ag/AgCl obtained with a CFM/Pt in deoxygenated phosphate-buffered solution (0.05 M, pH 7.4) at a scan rate of 0.05 V s^−1^ in the absence (a) and presence of increasing concentrations of H_2_O_2_ (b–e); 0.1–0.4 mM); Inset: plot of anodic (*I*_pa_) peak currents (at +0.7 V) as a function of H_2_O_2_ concentration, showing the respective linear regression equation.

The electrochemical behavior of the CFM/Pt microelectrodes was characterized by cyclic voltammetry in N_2_ flushed sulfuric acid solution. Cyclic voltammograms were recorded between −0.25 and +1.2 V vs. Ag/AgCl reference at increasing scan rates (50−1000 mV s^−1^) as shown in Figure 1A. The voltammogram can be divided into three regions: the underpotential deposition region of hydrogen (UPD-H) between approximately −0.25 and +0.2 V, where two well-defined peaks (at −0.04 and −0.14 V) evolve due to the adsorption/desorption of hydrogen atoms of the Pt surface; a flat current region due to the charging/de-charging of the electrical double layer (DL) close to the surface, between +0.05 and +0.3 V, and a redox peak region appearing at E > +0.6 V (oxidation) and around +0.5 V (reduction) in the subsequent negative-going sweep, due to the formation/reduction in Pt hydroxide/oxide at the Pt electrode surface [29,30,31].

The shape of the voltammogram is typical of bulk Pt in sulfuric acid solution, namely the hydrogen adsorption and desorption peaks and Pt oxide formation and reduction [32,33,34]. 

#### 2.2.2. Cyclic Voltammetry of Hydrogen Peroxide

The electrochemical response of CFM/Pt towards H_2_O_2_ oxidation can be observed in the cyclic voltammograms obtained in deoxygenated PBS (Figure 1B). In the presence of H_2_O_2_, the current starts increasing at +0.3 V in the forward sweep and in the backward sweep a reduction current peak appears at 0.0 V. It is noteworthy that there is a linear increase in current with increasing concentration of H_2_O_2_ (Figure 1B; inset) suggesting that the CFM/Pt microsensor can be used as a sensing platform for developing enzyme oxidase-based microbiosensors.

### 2.3. CFM/Pt-Based Lactate Microbiosensors

Measuring lactate changes in vivo in a complex biochemical environment, such as the brain extracellular space, requires the design of microbiosensors with high selectivity, sensitivity, and low response time, in addition to high spatial and temporal resolution, operational stability, minimal oxygen-, pH-, and temperature-dependencies, as well as suitable biocompatibility, and minimal tissue damage [35]. The merits of the CFM/Pt as a sensing platform for developing microbiosensors was assessed by using lactate oxidase immobilized on the tip surface of the CFM/Pt (Supplementary Materials, Figure S1). To extend the linear range, the microbiosensors were coated with an additional layer of polyurethane (PU). 

#### 2.3.1. Enzyme Kinetics and Analytical Performance

The analytical and enzyme kinetic parameters of the designed lactate microbiosensors were evaluated, and data are summarized in Table 1. The CFM/Pt microbiosensors were prepared by immobilizing lactate oxidase (LOx) with two enzyme loadings, via cross-linking with glutaraldehyde and BSA: 1 mg mL^−1^ (0.1%) and 5 mg mL^−1^ (0.5%) LOx, referred herein, respectively, as CFM/Pt-LOx(0.1%)/PU and CFM/Pt-LOx (0.5%)/PU. As expected, the PU layer increased the *K*_m,app_(L) from 1.5 ± 0.1 to 2.5 ± 0.3 mM and decreased the sensitivity from 4.5 ± 0.5 to 3.2 ± 0.6 nA mM^−1^. This effect is likely due to the diffusion restriction imposed by the PU membrane, which creates a diffusion barrier and limits the concentration of lactate that reaches the enzymatic matrix [36]. This decrease in sensitivity may also be due to the THF solvent used to prepare the PU solution that could partially inactivate the LOx enzyme.

The increase in LOx loading from 0.1% to 0.5% significantly increased microbiosensors sensitivity from 3.2 ± 0.6 to 10.8 ± 1.2 nA mM^−1^ and the maximum steady-state current (*I*_max_) from 9.3 ± 1.7 to 26.3 ± 3.9 nA, which is in accordance with the fact that this parameter gives an indication of the total amount of active enzyme immobilized onto the electrode surface [37]. The efficiency of the enzyme layer (%BE) was improved by increasing LOx loading in the matrix, but no significant effect was observed on the *K*_m,app_(L).

Taking these results into account, the CFM/Pt-LOx(0.5%)/PU biosensor was selected as the most appropriate design for lactate monitoring in vivo in the brain tissue, due to the higher *K*_m,app_(L) and sensitivity. 

The brain extracellular space is a complex chemical environment containing several electroactive molecules that can act as interferents in the measured amperometric current. In this regard, ascorbate (AA) is the major interferent due not only to the high basal concentration (ca. 400 µM) but also because this extracellular concentration can change significantly during neuronal activity [38,39]. Additionally, its oxidation potential at carbon or platinum electrodes is in range of +0.2 to +0.4 V vs. Ag/AgCl [39]. Therefore, the microbiosensors were coated with two Nafion^®^ layers to improve selectivity against major interferents. Nafion^®^ is a well-known negative charge polymer which repels anions, such as ascorbate [20,40,41]. The enzyme kinetics and analytical parameters of the Nafion^®^-coated biosensor were evaluated and are summarized in Table 1. 

**Figure 2:**
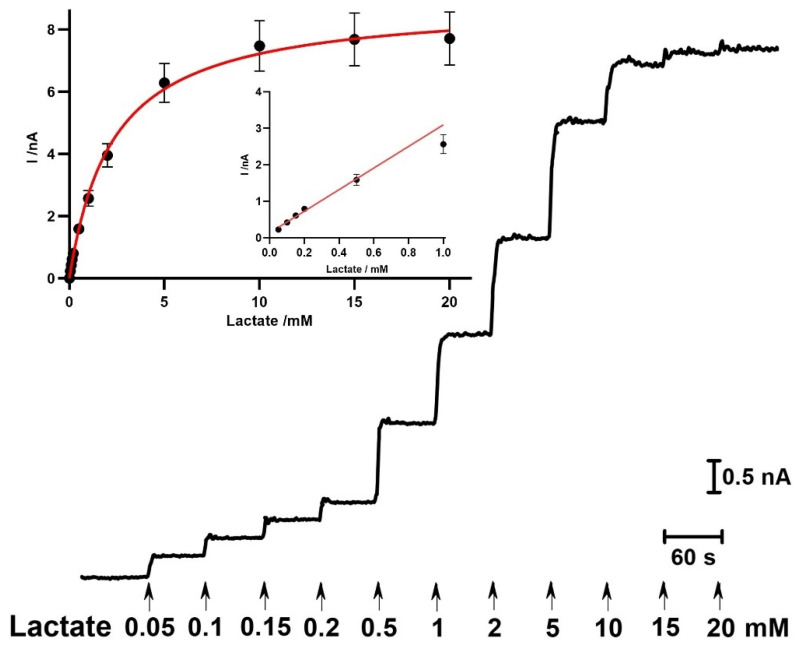
Enzyme kinetics of the CFM/Pt/Nafion^®^-LOx(0.5%)/PU biosensor. Representative calibration showing the response to successive additions of lactate, from 0.05 to 20 mM; Inset: calibration plot of the average steady-state current as function of lactate concentration. Data were fitted to the Michaelis–Menten equation by using non-linear regression analysis; Inset: linear regression of the biosensor response to lactate (y = 2.96x + 0.14; R^2^ = 0.992). Data represent mean ± SEM (*n* = 9).

A representative calibration of the CFM/Pt/Nafion^®^-LOx(0.5%)/PU showing the response to successive additions of lactate between 0.05 to 20 mM is shown in Figure 2. Data were fitted using a simplified one-substrate Michaelis–Menten model to determine the enzyme kinetic parameters and other analytical parameters including the upper limit of linearity (Figure 2: inset). 

Lactate microbiosensors exhibited analytical performance and enzyme kinetics that were comparable to other lactate microbiosensors previously described in the literature, as shown in Table 2. 

#### 2.3.2. Response Time

To record rapid fluctuations of lactate in vivo in the brain, the response time of microbiosensor must be within the seconds range. Current vs. time recordings for repeated injections of 1 mM lactate were used to calculate the response time (t_90%_–t_10%_) for the CFM/Pt/Nafion^®^-LOx(0.5%)/PU. On average, the microbiosensors showed a response time of 7.3 ± 1.1 s (*n* = 9) which allows a continuous monitoring of lactate flux between neurons and astrocytes in the brain extracellular space.

#### 2.3.3. Oxygen Dependency

In vivo monitoring can be affected by changes in oxygen concentration, temperature, and pH, which impact on the accuracy of lactate measurements. The dependency of O_2_, a co-substrate to the enzyme, is an important parameter to be evaluated [49]. Therefore, the response of CFM/Pt/Nafion^®^-LOx(0.5%)/PU microbiosensors to 0.5 and 1 mM of lactate was evaluated for variable O_2_ concentrations.

**Figure 3:**
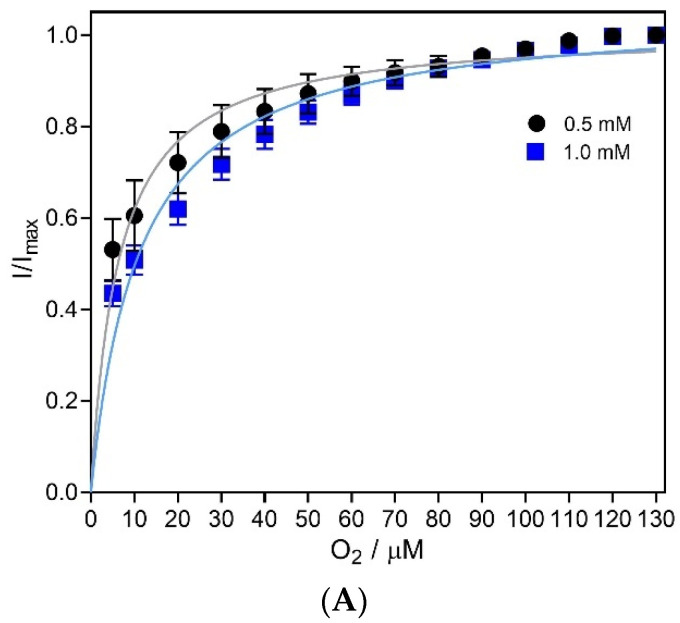

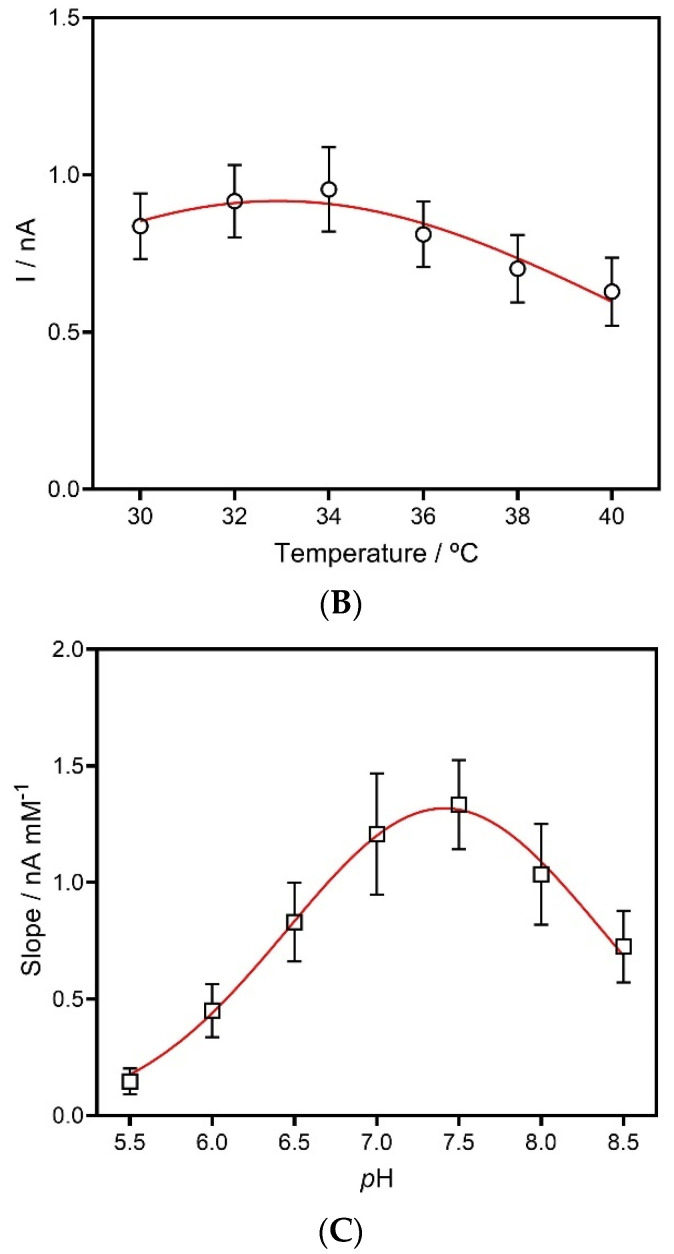
Evaluation of (**A**) oxygen, (**B**) temperature and (**C**) pH dependence of lactate microbiosensor. (**A**) Average normalized current detected by the CFM/Pt/Nafion^®^-LOx(0.5%)/PU biosensor to two lactate concentrations (0.5 and 1.0 mM) as function of oxygen concentration. (**B**) Effect of temperature on the recorded current of the CFM/Pt/Nafion^®^-LOx(0.5%)/PU biosensor in response to 0.5 mM of lactate. (**C**) Effect of pH on the sensitivity of CFM/Pt/Nafion^®^-LOx(0.5%)/PU biosensor towards lactate. Data represent mean ± SEM (*n* = 4).

The average steady-state current response to 0.5 and 1.0 mM lactate plotted as a function of O_2_ concentration followed a Michaelis–Menten kinetics model, as depicted in Figure 3A. For both lactate concentrations, the microbiosensor response was 80% of maximal response for O_2_ concentrations between 30 and 40 µM. Considering that the physiological O_2_ levels in the brain range from 30 to 50 µM [49,50], the microbiosensor in vivo performance is not expected to be limited by O_2_ availability. However, lower O_2_ concentrations, such as those observed under hypoxic and ischemic conditions [51], would be limiting.

#### 2.3.4. Effect of Temperature and pH

Considering that subtle changes in temperature and pH can occur in the brain extracellular space [52,53,54], and that these directly affect enzyme activity, these parameters were evaluated to fully characterize the lactate microbiosensor.

In terms of temperature dependency, the CFM/Pt-based microbiosensor response to the addition of 0.5 mM of lactate was measured in PBS, while varying the temperature between 30 and 40 °C (Figure 3B). A change of 1 °C from physiological temperature produced a current change between 1% and 3%.

Regarding the effect of pH, the microbiosensor sensitivity towards lactate and H_2_O_2_ was measured at 37 °C in PBS with pH ranging from 5.5 to 8.5. The obtained data displayed a bell-shaped response, with a maximum response at 7.4, which is in accordance with the pH levels reported for brain extracellular space (Figure 3C) [55]. A change of 0.1 from physiological pH that was expected to be observed in the brain extracellular space [55] produced a current change ca. 0.5%.

### 2.4. Concurrent Measurements of Lactate and Glucose

#### 2.4.1. Microbiosensor Dual Calibration

Multianalyte monitoring in the brain extracellular space is of major importance to better understanding neurometabolism and the dynamic interactions between metabolites such as lactate and glucose. In our previous work, we developed a CFM/Pt microbiosensor for ex vivo glucose measurements in brain slices [26], which we have combined here with the lactate microbiosensor for concurrent in vivo measurement of glucose and lactate.

**Figure 4:**
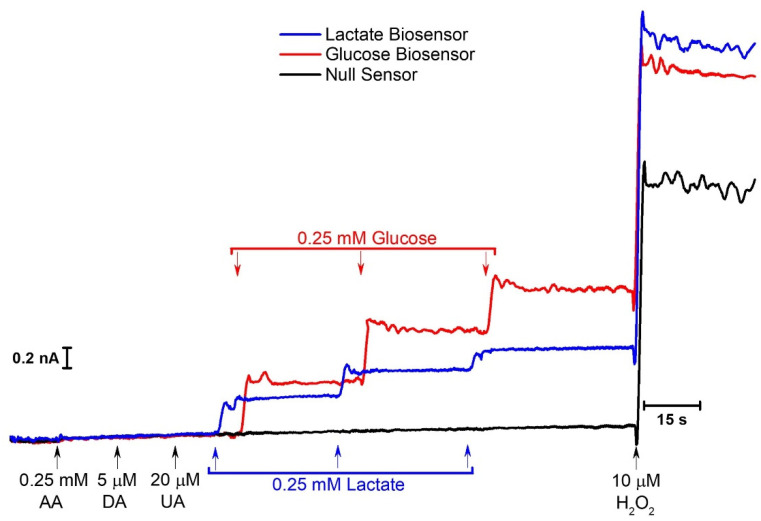
Representative recording of the calibration of the CFM/Pt/Nafion^®^-LOx(0.5%)/PU/PD and CFM/Pt/Nafion^®^-GOx(0.5%)/PD biosensor to lactate and glucose, respectively, in the presence of interferents. The recording shows the response of LOx coated (blue), GOx coated (red), and null CFM/Pt (black) to 0.25 mM of ascorbate, 10 µM of dopamine, 20 µM of uric acid, three consecutive additions of 0.25 mM of lactate and glucose, and 10 µM of H_2_O_2_.

To further increase selectivity, microbiosensors were modified by electrodeposition of *m*-PD which acts as a permselective membrane and as a molecular filter for molecules, such as ascorbate and dopamine, limiting their access to the electroactive surface, while allowing the access of small molecules, such as H_2_O_2_ [56]_._ A representative dual calibration of the microbiosensors towards lactate and glucose in the presence of major interferents is presented in Figure 4.

The responses to ascorbate (0.25 mM), dopamine (5 µM) and urate (20 µM) are almost negligible compared to that of lactate (0.25 mM), glucose (0.25 mM), and H_2_O_2_ (10 μM), resulting in a Lactate:Interferent selectivity on a molar ratio of 29.8 ± 0.7:1 (*n* = 8), 6.2 ± 0.3:1 (*n* = 8), and 4.3 ± 0.5:1 (*n* = 7) for ascorbate, dopamine, and urate, respectively. The corresponding blocking efficiency was 97% for ascorbate, 84% for dopamine, and 77% for urate.

#### 2.4.2. In Vivo Monitoring of Neurometabolic Markers in the Rat Brain

We have implanted in a rat brain an array configuration comprising the lactate and glucose microbiosensors and the null sensor glued to a glass micropipette to locally application of solutions, to assess and validate its recording capabilities in vivo (Supplementary Materials, Figure S2).

In a first set of experiments, the array was inserted in the brain cortex or hippocampus and the background current was allowed to stabilize for at least 30 min. For each brain region, basal lactate and glucose concentration were estimated by subtracting the null sensor current from that of each microbiosensor. Basal lactate and glucose concentrations in the cortex were 0.64 and 0.79 mM, while in the hippocampus they were 0.68 and 2.3 mM, respectively. These values are consistent with those reported in other in vivo studies using other types of biosensors in which brain lactate and glucose range from 0.3 to 2 mM [47,57,58].

**Figure 5:**
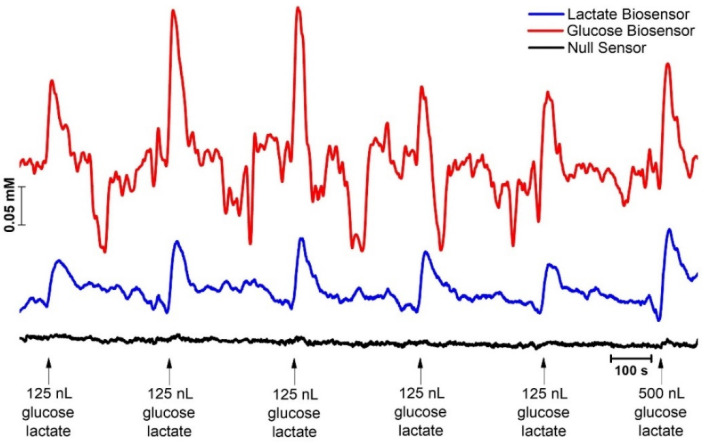
In vivo recording of lactate (blue) and glucose (red) in the cortex (AP: −4.0; ML: −2.5; DV: −1.5). Black line represents current recorded at the null sensor. Arrows indicate moment of pressure ejection (1 s, 20 PSI) of a sodium lactate and glucose solution (0.1 mM) from a micropipette placed at 150 µm from the recording microelectrodes. Current to concentration conversion was accomplished with resource to pre-calibration sensitivity, since no significant loss in sensitivity was observed in the post-experiment calibration performed after 4 h of implantation in brain tissue.

We then evaluated the response to local application of a solution containing lactate and glucose delivered through the micropipette. As can be observed in Figure 5, a rapid and transient increase in current was observed at both the glucose and lactate microbiosensors in response to pressure injection of the solution. This supports the suitability of both microbiosensors for monitoring rapid changes in brain lactate and glucose concentrations.

We then investigated the effect of changing blood glucose levels on hippocampal levels of lactate and glucose. For this purpose, hyperglycemia was induced by systemic administration of glucose (1.6 g kg^−1^, i.p.), followed by administration of insulin (10 U kg^−1^, s.c.) to induce hypoglycemia. Blood glucose concentration was measured every 10 min with a commercial glucose monitor and test strip.

**Figure 6:**
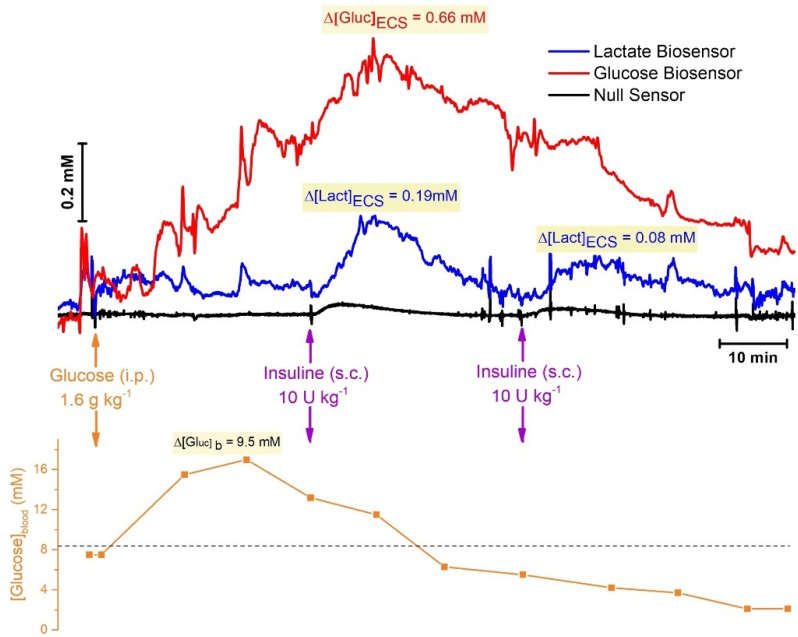
**Top Panel:** In vivo monitoring of lactate (blue) and glucose (red) in the brain extracellular space (hippocampus, AP: −3.5; ML: −2.5; DV: −2.9). Black line represents current recorded at the null sensor. **Bottom Panel:** Systemic glucose concentration measured in 10 min intervals using a commercial glucose meter and blood collected from tail vain. Dotted line represents basal blood glucose concentration. Arrows indicate moment of system administration of glucose (1.6 g kg^−1^, intraperitoneal) and insulin (10 U kg^−1^, sub-cutaneous). Shade boxes highlight maximal variation in the concentration of each analyte measured.

As can be observed in the representative recording shown in Figure 6, glucose administration induced a rapid increase in blood glucose levels (maximum increase of 9.5 mM, 25 min) which was followed by an increase in the brain glucose concentration (maximum increase of 0.66 mM, 40 min). We hypothesize that, the increase in brain glucose concentration was the result of transport across the blood–brain barrier (BBB) by type 1 glucose transporters (GLUT1) in a facilitated diffusion mechanism [58] that is insensitive to insulin, and conveys about 50 times more glucose into the central nervous system than would otherwise enter [59]. Despite the increase in the brain glucose levels, no change in lactate was observed during this hyperglycemic phase. Following insulin administration, blood glucose concentration decreased continuously throughout the remainder of the recording, falling below initial baseline values. In brain tissue, the decrease in glucose was slower than that observed for blood and remained above baseline values. The effect of insulin administration on blood and brain extracellular glucose concentration was in agreement with other studies carried out with microbiosensors [57,60,61,62,63].

Furthermore, insulin induced a transient increase in brain lactate levels (0.19 mM and 0.08 mM for the first and second insulin administration, respectively). The local increase in extracellular lactate in response to insulin may be the result of either an increased uptake from circulation or an increased production in brain tissue. Lactate levels in circulation have been demonstrated to fluctuate synchronously with insulin in humans [64]. Furthermore, an increase in plasma lactate levels in normal nondiabetic subjects following an hyperinsulinemic euglycemic clamp has also been reported, suggesting that insulin per se evokes increased lactate production, independent of glycemic level [65]. The transient increase in brain extracellular lactate following insulin administration possibly reflects stimulation of the glycolytic metabolism of glucose producing pyruvate that is further converted to lactate.

It is important to note that insulin can cross the BBB and has been shown to be trans-ported by a saturable system. Most relevantly, insulin uptake rate and relative distribution in brain tissue is not uniform, and the hippocampus is amongst one of the regions with highest uptake of systemic insulin [66]. Thus, we cannot exclude that the increase in local lactate is the direct effect of insulin acting on neural cells, namely astrocytes which are amenable to increased glycolysis through the regulation of 6-phosphofructo-2-kinase/fructose-2,6-biphosphatase [67].

The changes in the levels of lactate and glucose measured in this study are consistent with previous reports [36,46,47,64,65], demonstrating that the biosensors based on CFM/Pt platforms can be used for continuous monitoring the changes in these neurometabolic substrates in vivo in the brain extracellular space. Furthermore, they can potentially be combined with previously describe O_2_ microelectrodes [66,68]. Future work should include the addition of a pyruvate microbiosensor, which would allow a deeper understanding of the interplay between these metabolic substrates and brain activity.

## 3. Materials and Methods

### 3.1. Chemicals and Solutions

All chemicals were of analytical grade and used as received. The hydrogen hexachloroplatinate solution (8% in H_2_O), lactate oxidase (LOx) from *Aerococcus viridians* in lyophilized powder form, glucose oxidase (GOx) from *Aspergillus niger* in powder form, bovine serum albumin (BSA), glutaraldehyde solution (25%), sodium L-lactate, D-(+)-glucose, hydrogen peroxide solution (30% *w/w*), Nafion^®^, polyurethane (PU), tetrahydrofuran (THF), dimethylformamide (DMF), *meta*-phenylenediamine (m-PD), ascorbate, dopamine, urate, and urethane were obtained from Sigma Aldrich, Portugal. Insulin was obtained from Lilly, Portugal. Unless otherwise stated, all solutions were prepared in bi-deionized MilliQ water with resistivity ≥18 MΩ cm (Millipore Corporation, Burlington, MA, USA). Argon and oxygen were provided by Air Liquide, Portugal. The electrolyte for in vitro analytical evaluation of microbiosensors was phosphate-buffered saline (PBS lite) 0.05 M (pH 7.4) with the following composition (mM): 100 NaCl, 10 NaH_2_PO_4_ and 40 Na_2_HPO_4_. The solution of H_2_PtCl_6_ 0.4% was prepared in 0.1 M H_2_SO_4_. The working solutions of H_2_O_2_ (9.8 mM) and ascorbate (20 mM) were prepared freshly each day. Stock solutions of lactate (1 M), glucose (1 M), dopamine (20 mM), and urate (20 mM) were prepared and conserved at 4 °C protected from light.

The solutions used for intracranial pressure-ejections were prepared in saline (0.9% NaCl), adjusted to pH 7.4, and filtered prior to use (0.2 µm pore size filter).

Saturated O_2_ solutions for oxygen dependence evaluation were prepared by bubbling PBS lite with 100% O_2_ at 37 °C for 20 min, resulting in an O_2_ solution at 722 mmHg (1.0 mM concentration [69]).

### 3.2. Electrochemical Instrumentation

A MultiPalmSens IV (PalmSens BV, Houten, The Netherlands) potentiostat controlled by MultiTrace v 4.2 software was used for chronoamperometric and voltametric measurements. A three-electrode electrochemical cell was used comprised of the working electrode, an Ag/AgCl (3 M NaCl) reference electrode (RE-5B) and a Pt wire as an auxiliar electrode. Amperometric calibration and recording were performed using a FAST16mkII potentiostat (Quanteon, Nicholasville, KY, USA) in a two-electrode electrochemical cell configuration mode comprised of the working electrode and Ag/AgCl (3 M NaCl) reference electrode. For in vivo recordings, an Ag/AgCl wire reference electrode was used by electro-oxidation of the exposed tip of a Teflon-coated Ag wire (200 μm o.d., Science Products GmbH, Hofheim, Germany) in 1 M HCl saturated with NaCl, which develops an Ag/AgCl half-cell when in contact with cerebrospinal fluid in the brain containing chloride ions.

### 3.3. Fabrication of Carbon Fiber Microelectrodes

Carbon fiber microelectrodes were fabricated, as previously described [70]. Briefly, single carbon fibers (30 μm Textron Lowell, Wilmington, MA, USA) were inserted into borosilicate glass capillaries (1.16 mm i.d. and 2.0 mm o.d., Harvard Apparatus, Holliston, MA, USA) and cleaned with acetone. Each capillary was pulled on a vertical puller (Harvard Apparatus, UK). The protruding carbon fibers were cut to a tip length of 150–250 μm. The electrical contact between the carbon fiber and the copper wire was provided by conductive silver paint (RS, Northants, UK). As a result, the obtained microelectrodes presented a cylindrical geometry, with 30 µm in diameter and with a tip length in the range of 150 to 250 μm. The microelectrodes were tested for general recording properties in PBS by fast cyclic voltammetry at a scan rate of 400 V s^−1^, between −0.4 and +1.6 V vs. Ag/AgCl. Microelectrodes deemed acceptable for recording was based on the stability of the background current and sharp transients at reversal potentials. All CFMs were clean with isopropanol before the platinization process.

### 3.4. Carbon Fiber Microelectrode Platinization

The exposed carbon tip surface of bare CFM was modified by electrodeposition of Pt, as described in [26]. Briefly, the electrodeposition of Pt onto the carbon surface was carried out by using a deoxygenated 0.4% chloroplatinic acid solution in 0.1 M H_2_SO_4_ in a two-electrode electrochemical cell (MultiPalmSens4 Potentiostat, PalmSens, Houten, The Netherlands). Potentiostatic electrodeposition was performed by applying a holding potential of −0.2 V vs. Ag/AgCl for 10 s. By integrating the amperometric current during the 10 s of platinum electrodeposition, the total amount of material deposited on the microelectrode according with the Faraday Laws was 6.97 ± 0.18 × 10^−8^ g (*n* = 10). The platinization process was evaluated by measuring the amperometric current at +0.7 V vs. Ag/AgCl following four successive additions of H_2_O_2_.

### 3.5. Immobilization of Lactate Oxidase

Lactate oxidase was immobilized onto the Pt-modified CFM surface (CFM/Pt) by crosslinking with glutaraldehyde (GA) in the presence of BSA (protective agent), as described previously [26]. To obtain the lactate microbiosensor (CFM/Pt-Lox) the tip of the CFM/Pt was steeped for 5 min in a drop of a cocktail solution containing either 1.0 or 5.0 mg mL^−1^ of Lox, 10% BSA and 0.125% GA in water. The procedure was repeated three times with 1 min of interval. Null or sentinel sensors (CFM/Pt-Null) to be used in self-referencing recordings were obtained by coating the CFM/Pt tip surface with an inactive protein matrix solution (BSA and GA) in the absence of Lox and following the same procedure. Both CFM/Pt-Lox and CFM/Pt-Null were stored dry at room temperature, protected from light and dust, for at least 72 h for curing.

The microbiosensors were further coated with a layer of polyurethane (PU) to extend the linear range for the substrate detection and to protect the enzyme layer from biofouling in the brain tissue. The tips of the CFM/Pt-Lox and CFM/Pt-Null were dipped three times in a 2% solution of PU dissolved in a THF and DMF solution (98% and 2% *v/v,* respectively) with 10 min intervals between dips at room temperature to dry the layers (CFM/Pt-LOx/PU; CFM/Pt-Null/PU). To minimize the interference of electroactive compounds, such as ascorbate, and consequently enhance selectivity towards lactate, Nafion^®^ and m-PD layers are included in the developed electrodes designs. After the platinization procedure, the microelectrodes are oven-dried for 5 min at 170 °C to remove traces of humidity. Then, the microelectrodes are coated with Nafion^®^ by dipping the tip of the carbon fiber into a fresh Nafion^®^ solution (5% in aliphatic alcohols) at room temperature for 5 s and drying in an oven for 15 min at 170 °C. The process is repeated to attain two Nafion^®^ layers. Further, the sensors surface is modified with an exclusion layer of *m*-PD. Briefly, 5 mM *m*-PD in deoxygenated PBS was electropolymerized by CV at a scan rate of 50 mV s^−1^, between +0.25 and + 0.75 V vs. Ag/AgCl for 20 min (FAST MKII, Quanteon LLC, Nicholasville, KY, USA).

### 3.6. In Vitro Evaluation and Characterization

Microelectrodes were evaluated for their response towards lactate and H_2_O_2_ by amperometry at + 0.7 V vs. Ag/AgCl in a two-electrode configuration mode (FAST16mk-II, Quanteon LLC, Nicholasville, KY, USA). The enzyme analytical and kinetic parameters regarding lactate measurements were determined in 0.05 M PBS at 37 °C under moderate stirring by evaluating the response towards consecutive additions of lactate (final concentration range: 0.05–20 mM). The sensitivity to lactate, selectivity against major interferents, and the sensitivity to the reporter molecule were determined by three additions of lactate (final concentration range: 0.25–0.75 mM) in the presence of ascorbate 0.25 mM, dopamine 10 µM, and urate 20 µM, followed by H_2_O_2_ 10 µM. The sensitivity towards H_2_O_2_ was determined in PBS 0.05 M at room temperature under moderate stirring by evaluating the response to four consecutive additions of H_2_O_2_ stock solution (final concentration range: 10–40 μM). The stability of the CFM/Pt-based LOx biosensors was evaluated at the end of acute brain implantation.

To evaluate the oxygen dependence of the CFM/Pt-based LOx biosensors, the response to lactate was measured under variable oxygen concentrations. The O_2_ dependence experiment was carried out in 40 mL PBS 0.05 M at 37 °C under gentle stirring after a period of current stabilization. Then, lactate (0.5, 1.0 mM) was added to the beaker. After stabilization, the PBS solution was purged with N_2_, to withdraw the O_2_. Then, constant volumes of the O_2_ saturated solution were added to the solution. To evaluate the effect of temperature, the response to 0.5 mM of lactate was measured in PBS (pH 7.4), while varying the temperature between 30 °C and 40 °C. To test the effects of pH the sensitivities to lactate was evaluated at 37 °C in PBS, with pH ranging from 5.5 to 8.5.

### 3.7. Surgical Procedures and In Vivo Experiments

All the procedures used in this study were performed in accordance with the European Union Council Directive for the Care and Use of Laboratory animals (2010/63/EU) and were approved by the local ethics committee (ORBEA). The present in vivo studies were carried out on adult male Wistar rats (8–10 weeks, weight between 250 and 350 g) maintained in controlled environmental conditions: temperature of 22–24 °C, relative humidity of 45–65%, 15 air exchanges per hour, and a 12:12 light/dark cycle. Animals were housed in filter-topped type III Makrolon cages on an individually ventilated caging system (VentiRack BioscreenTM, Margate, UK). Rats were fed with a standard rat chow diet (4RF21-GLP Mucedola, SRL, Settimo Milanese, Milan, Italy) and were provided with chlorinated water, available in ad libitum. The experimental setup used for recording lactate in vivo in anesthetized rats was similar to the one used in previous studies [41,70,71].

Briefly, rats were anesthetized with urethane (1.25–1.50 g/kg^−1^, i.p.) and placed in a stereotaxic frame (Stoelting, Wood Dale, IL, USA). Animal temperature was maintained at 37 °C by using a deltaphase isothermal pad (Braintree Scientific, Braintree, MA, USA). After exposing the skull by a midline scalp incision, a hole was drilled in the skull overlying the brain area of interest. The meninges were refracted prior to the insertion of the microbiosensor array into the rat brain according to coordinates calculated from bregma based on the rat brain atlas of Paxinos and Watson [72]. Specific coordinates used in each experiment are indicated in each figure legend. A small hole was drilled in a site remote from the recording area and a miniature Ag/AgCl pseudo-reference electrode was inserted and soaked with a 0.9% NaCl solution during the experiments. The micropipette was filled with a flexible microfilament (MicroFil, World Precision Instruments, Hitchin, UK). Amperometric recordings were started, and the background currents were allowed to stabilize for at least 30 min. Once the background currents were stable, solutions were locally pressure ejected into the brain adjacent to the microbiosensor from the glass micropipette using a Picospritzer III (Parker Hannifin Corp., General Value, Fairfield, NJ, USA). Insulin (10 U kg^−1^) and glucose (1.6 g kg^−1^) were administered sequentially via subcutaneous and intraperitoneal injection, respectively. Blood glucose concentration was measured every 10 min with a commercial glucose monitor and test strip (FreeStyle-Precision Neo, Abbot) with blood collected from tail vain. To assess the operation stability, the response of the lactate biosensors was tested before and after the in vivo experiments. The microbiosensor sensitivity slightly de-creased in the post-calibration (6 ± 1.3%, *n* = 4).

### 3.8. Data Analysis

Data analysis was performed using GraphPad Prism 5.0 and OriginPro 2016. The sensitivity towards H_2_O_2_ was determined by linear regression between 10 and 40 μM. Enzyme kinetic analysis of the microbiosensor response to lactate was performed by fitting calibration data to a Michaelis–Menten type equation form in which the maximum steady-state current (*I*_max_) and apparent Michaelis–Menten constant for lactate (*K*_m,app_(L)) were calculated. The sensitivity in the linear region (LRS) was determined by linear regression analysis in a range of 0–0.5 mM. The selectivity towards interferents (ascorbate, dopamine, uric acid), on a molar basis, was calculated as the ratio of the sensitivities for lactate and interferents. The limit of detection (LOD) was defined as the concentration of analyte corresponding to a signal-to-noise ratio of 3. The response time (t_90%_–t_10%_) of the microbiosensors was determined by fitting a Boltzmann sigmoid function and calculating the time interval between 10% and 90% of the lactate response. Values are given as the mean ± standard error of the mean (SEM).

## 4. Conclusions

In the present work we describe the design and evaluation of a lactate microbiosensor for monitoring basal and transient changes of lactate in brain extracellular space with high spatial and temporal resolution and high analytical performance. Carbon fiber microelectrode modified with electrodeposited Pt nanoparticles were used as a sensing platform for the construction of lactate oxidase-based microbiosensor, based on cross-linking of LOx with glutaraldehyde on the CFM/Pt surface. Addition of permselective membranes (Nafion^®^ and PD) increased selectivity while coating with PU extended the microbiosensor linear range to values suitable for in vivo monitoring. The in vitro evaluation of microbiosensors showed good analytical properties for measuring lactate with high sensitivity and selectivity and low dependence on oxygen, temperature, and pH.

The novel lactate microbiosensor was combined with a glucose microbiosensor previously designed by us to perform concurrent monitoring of both neurometabolic substrates in vivo in the rat brain. Having demonstrated the feasibility of this approach by successfully monitoring locally applied glucose and lactate to the brain, we determined basal levels of both metabolites in the cortex and hippocampus, taking advantage of a null sensor for self-referencing.

Finally, the microbiosensors were used to explore the interplay between glucose and lactate in the hippocampus by modulating blood glucose levels with insulin, revealing that the influx of glucose to the brain responds to changes in systemic glucose levels and that insulin induces a transient increase in local lactate levels. Future work will include the design of a pyruvate microbiosensor to combine with the glucose and lactate biosensors, as well as the addition of an O_2_ microsensor. The concurrent real-time monitoring of these neurometabolic markers is a valuable tool to investigate the neurometabolism and to better understand the played role of major neurometabolic markers in vivo.

## Figures and Tables

**Table 1:** Enzyme kinetics and analytical parameters of the CFM/Pt-based microbiosensors.

Type (N)	*K*_m,app_(L) (mM)	Sensitivity (nA mM^−1^)	*I*_max_ (nA)	LOD (µM)	%BE
CFM/Pt-LOx(0.1%) (8)	1.5 ± 0.1	4.5 ± 0.5	7.5 ± 0.9	10.2 ± 1.2	2.5 ± 0.1
CFM/Pt-LOx(0.1%)/PU (8)	2.5 ± 0.3	3.2 ± 0.6	9.3 ± 1.7	18.9 ± 2.4	4.2 ± 0.5
CFM/Pt-LOx(0.5%)/PU (6)	2.2 ± 0.3	10.8 ± 1.2	26.3 ± 3.9	10.0 ± 1.8	8.7 ± 3.0
CFM/Pt/Nafion^®^-LOx(0.5%)/PU (9)	2.3 ± 0.2	3.9 ± 0.4	8.9 ± 1.1	11.1 ± 1.5	1.1 ± 0.3

**Table 2:** Enzyme kinetics and analytical parameters of lactate microbiosensors.

Electrode type	*K*_m,app_ (mM)	*I*_max_ (nA)	Sensitivity (nA mM^−1^)	L. Range (mM)	LOD (µM)	Ref.
CFM/Pt/Nafion^®^-LOx(0.5%)/PU	2.3	8.9	3.9	0.05–0.5	11.1 ± 1.5	Present Study
CFM/LOx/CA	-	-	9.15 (nA M^−1^ cm^−2^)	0.1–2.0	-	[42]
CFM/PB/LOx/PoPD	0.44	2.88	2.68	0.0056–0.6	5.6	[43]
CFM/PB/LOx/PoPD/Nafion^®^	0.97	1.57	0.77	0.0168–1.2	16.8	
CFM/Chit-LOx	-	-	22	0–1	7.0	[44]
CFM/Ru/LOx/Nafion^®^-PU	0.48	54	-	0–1.75	2	[36]
CFM/Ti-Pt/LOx/PU	2.60	-	0.20 nA (nA µM^−1^ mm^−2^)	-	1.16	[18]
MEA/LOx-PU	22	-	3.0 (µA µM^−1^ cm^−2^)	0.05–5	10.6 ± 2.2	[45]
MEA/Nafion^®^/LOx/PU	-	-	0.089	2–20	78 ± 13	[46]
Ptwire/PmPD/LOx(0.2)/PAN	2.26	544.5	241.14	0.02–1.13	0.86 ± 0.21	[47]
Ptwire/Ir/p-OPD/LOx/PU	8.7	63.6	4.16	0–10	-	[48]

CA: cellulose acetate; PB: Prussian Blue; PoPD and p-oPD: poly(*o*-phenylenediamine); Chit: Chitosan; MEA: microelectrode array; PmPD: poly(*m*-phenylenediamine); PAN: Polycarilonitrile.

## Data Availability

The data presented in this study are available on request from the corresponding author.

## Supplementary Materials

The following supporting information can be downloaded, Figure S1: Schematic representation of the CFM/Pt-based LOx biosensor. The platinized carbon fiber was coated with a cocktail solution containing 1 or 5 mg/mL Lactate Oxidase (green dots), 10% BSA (yellow dots) and 0.125% glutaraldehyde, in water.; Figure S2: Photo and schematic representation (lateral and top view) of the microbiosensors array comprising the lactate and glucose microbiosensors and the null sensor glued to a glass micropipette.
