# Supplementary material for: Design and Evaluation of a Lactate Microbiosensor: Toward Multianalyte Monitoring of Neurometabolic Markers In Vivo in the Brain
Source: Molecules. 2022 Jan 14;27(2):514. doi: 10.3390/molecules27020514 (PMC8780383; doi:10.3390/molecules27020514)
Supplement: Supplementary file 1 [file molecules-27-00514-s001.zip › molecules-1511635-supplementary.pdf]

Supplementary Materials

Design and Evaluation of a Lactate Microbiosensor: Toward Multianalyte Monitoring of Neurometabolic Markers *In Vivo* in the Brain

Eliana Fernandes [1,2], Ana Ledo [1,2] and Rui M. Barbosa [1,2,*]

[1] Faculty of Pharmacy, University of Coimbra, 3000-548 Coimbra, Portugal; eliana.fernandes2604@ff.uc.pt (E.F.); analedo@ff.uc.pt (A.L.)
[2] Center for Neuroscience and Cell Biology, University of Coimbra, 3004-504 Coimbra, Portugal
* Correspondence: rbarbosa@ff.uc.pt

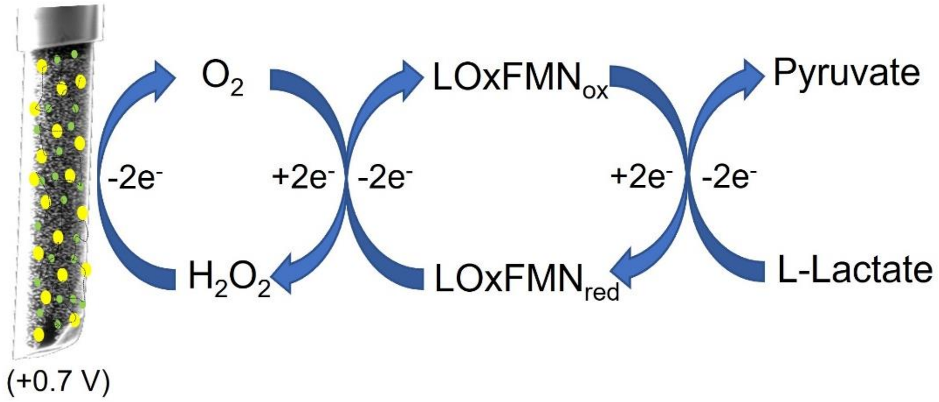

Figure S1 – Schematic representation of the CFM/Pt-based LOx biosensor. The platinized carbon fiber was coated with a cocktail solution containing 1 or 5 mg/mL Lactate Oxidase (green dots), 10% BSA (yellow dots) and 0.125% glutaraldehyde, in water.

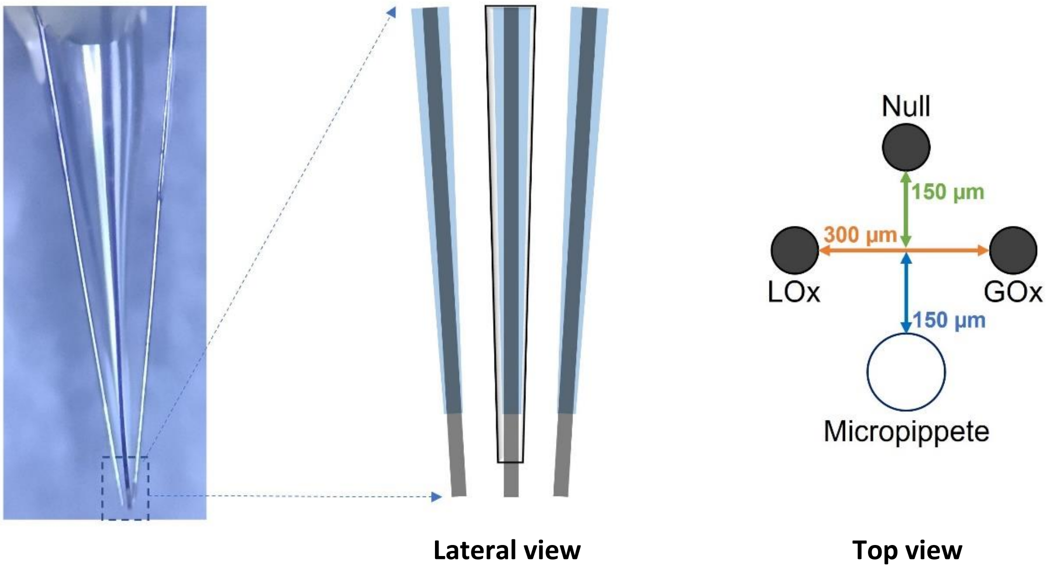

Figure S2 – Photo and schematic representation (lateral and top view) of the microbiosensors array comprising the lactate and glucose microbiosensors and the null sensor glued to a glass micropipette.
